# The prototype BS-II for computer measurement of biomechanical characteristics of the human cadaverous lumbar spine

**DOI:** 10.1186/s13018-019-1463-8

**Published:** 2019-12-19

**Authors:** Vladislav Janák, Luděk Bartoněk, Lumír Hrabálek, Jiří Keprt, Jiří Charamza

**Affiliations:** Univerzita Palackeho Prirodovedecka Fakulta, Olomouc, Czech Republic

**Keywords:** Lumbar spine, Strain gauge, LabVIEW, DIP, Lumir XLIF CAGE, BS-II

## Abstract

**Background:**

The new second-generation computer system BS-II (Bio-Spine-II) based on the National Instruments’ development environment has been designed and constructed for evaluating the stability of various surgical fixative methods of the cadaverous lumbar spine (L1–L5). BS-II holds the measured sample using aluminum fixtures and using four computer-controlled stepper motors; it performs a circular movement (warm up of the specimen), programmatically driven extension (back bend), right and left lateral flexion (lateral bend), left and right axial torsion (rotation), and axial compression (pressure). Four strain gauges are used to measure the stiffness of the sample. The movement of individual components (vertebrae) is contactlessly monitored by two CCD (charge couple device) cameras. The obtained data are in digital form continuously stored in the computer memory for further processing.

**Methods:**

The functionality of the equipment was verified on the cadaverous specimen of the human spine. The stiffness of the sample was measured by strain gauges, and the results were processed using linear regression analysis. Movements of vertebrae were determined by circular discs covered with appropriate patterns. The discs have been linked with the respective vertebrae and were contactlessly monitored by two CCD (charge couple device) cameras and evaluated using digital image processing methods and 2D digital FFT (fast Fourier transformation). Direction and displacement of the individual components were determined by the band of the calculated spectrum. The new device BS-II is controlled by a modifiable computer program designed in the National Instruments’ development environment which allows statistical processing of the sample, including its warming up.

**Results:**

The computer system BS-II for measurement of biomechanical properties of the spine sample was designed. Functionality of the device was verified by implementation of LUMIR XLIF CAGE implant into a cadaver sample of the human spine. Comparison of the rigidity of the intact and stabilized sample is shown in the graphs of article. The achieved results contributed to certification of the implant into the surgical practice.

**Conclusion:**

The designed computer BS-II system is designed for biomechanical measurements of the lumbar part of the human spine, especially for verification of surgical fixation methods. The system is based on the knowledge and experience with a manually operated measuring device designed by Palacky University Olomouc. The computer programmatic control allows the user to change the conditions and parameters of the measurement procedure in a planned way, which allows the results to be processed in, among other things, a statistical way.

If suitable models are used (3D printing), the BS-II system can be used to verify procedures for surgical stabilization of the spine in the training of future doctors.

The obtained data of stiffness and image information are stored in digital form and can be used for next offline sophisticated study of biomechanical properties of specimens (accurate vertebral geometry, statistical processing, 3D printing, etc.).

The usefulness of the BS-II system is demonstrated in the case of biomechanical analysis of the implantation of LUMIR XLIF CAGE implant to a human cadaver specimen of the spine.

## Introduction

**Fig. 1:**
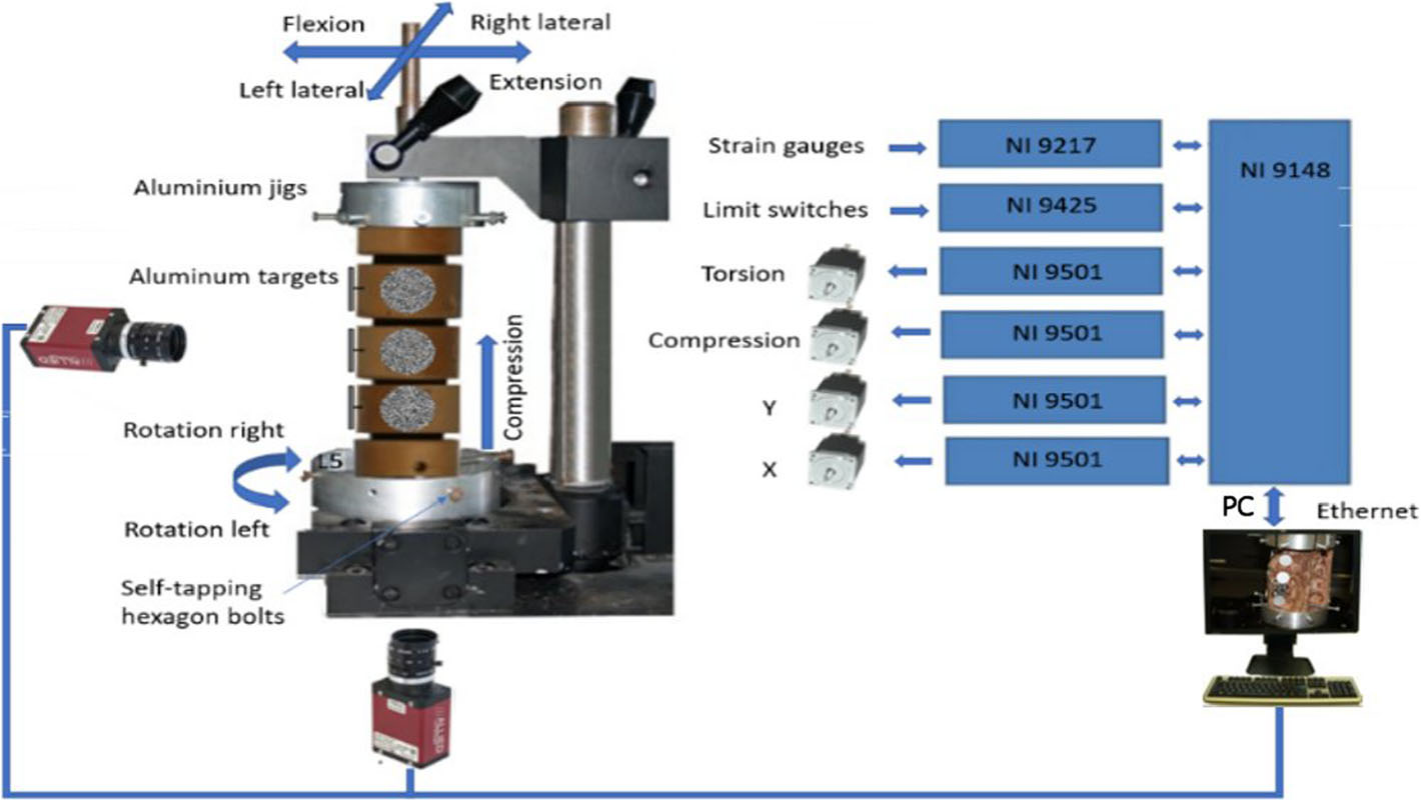
Block diagram of the measuring device BS-II

From the third decade of age, up to 80% of the population is suffering degenerative changes in individual spinal sections, particularly in the lumbar spine. The result of these changes is a pathogenic increase in the mobility of the vertebrae. According to Panjabi [1], the said spine instability is defined as a reduced backbone’s ability to respond to physiological stress. Spinal instability requires surgical stabilization—fixation of the affected motion segment. Different methods of instrumentation have been used for this if we are talking about the so-called internal fixation. In anterior access by the anterior lumbar interbody fusion (ALIF) or extreme lateral interbody fusion (XLIF), a bone autotransplant or titanium cage is added to the intervertebral disc after the intervertebral disc has been removed to provide interbody fusion. The constructed prototype BS-II allows comparison of the instant stiffness of the intact cadaveric specimen of the spine with the stabilized sample in six types of stress and the recording of the coordinates of its individual components by two cameras displaced by 90°. See Fig. 1.

## Methods

**Fig. 2:**
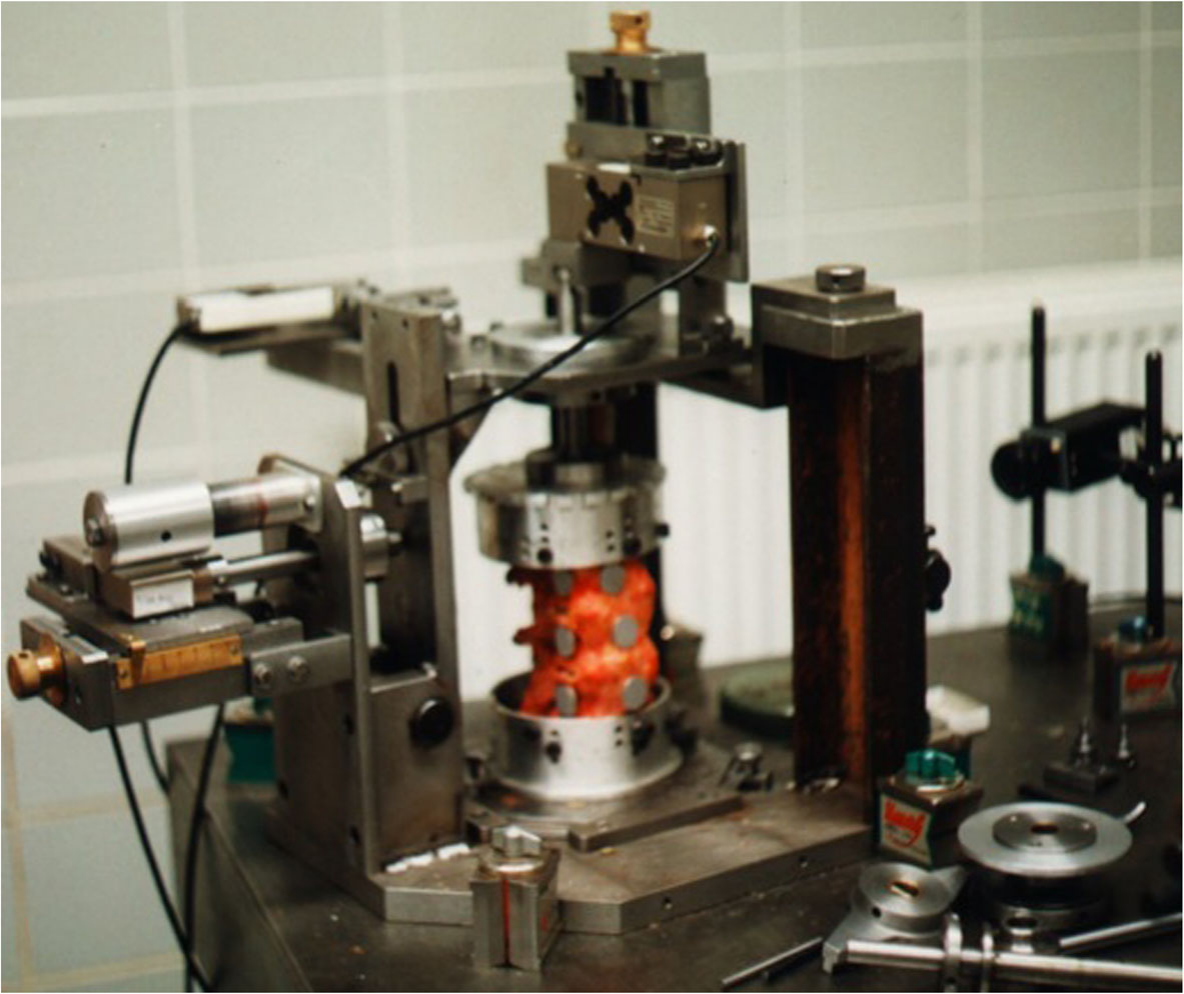
Biomechanical device of the type I (manual control)

The system of measuring the biomechanical characteristics of lumbar spinal samples was derived from the published article [13], which had been realized on a special (manually operated) device designed for this purpose (Fig. 2).

Experimental measurement methods were derived from [1] and [12, 13], where the biomechanical properties of the sample are characterized by the so-called immediate rigidity.

A rigidity of axial compression *R*_*c*_ was defined as the ratio of axial force (load) *F* and axial change of the length ∆*l* of the specimen

1 $$ {R}_c=\frac{F}{\varDelta l};\kern0.5em \left[{R}_c\right]=\frac{N}{mm}. $$

A rigidity of axial torsion can be expressed as the ratio of axial torque moment and the angle of rotation *ϕ* (Eq. 2a or, in a simplified form, Eq. 2b):

2 $$ 2a\left)\kern0.5em {R}_{{\mathrm{t}}_1}=\frac{Fr}{\varphi };\left[{R}_{{\mathrm{t}}_1}\right]=\frac{\mathrm{Nm}}{\mathrm{rad}},\kern0.5em 2b\right)\kern0.5em {R}_{{\mathrm{t}}_2}=\frac{Fr}{d};\left[{R}_{{\mathrm{t}}_2}\right]=\frac{\mathrm{Nm}}{\mathrm{mm}}, $$

where *ϕ* is replaced with the shift *d* of the constant arm *r*.

A rigidity of the sagittal flexion and extension and lateral bending tests are both evaluated as the ratio of bending moment and the angle of flexion (extension, bending), see Eq. 3a or, in simplified form, Eq. 3b:

3 $$ 3a\left)\kern0.5em {R}_{{\mathrm{f}}_1}=\frac{Fl}{\alpha };\left[{R}_{{\mathrm{f}}_1}\right]=\frac{\mathrm{Nm}}{\mathrm{rad}},\kern0.5em 3b\right)\kern0.5em {R}_{{\mathrm{f}}_2}=\frac{Fl}{d};\left[{R}_{{\mathrm{f}}_2}\right]=\frac{\mathrm{Nm}}{\mathrm{mm}}, $$

where also the angle *α* is replaced with the shift *d* of the arm *l*.

**Fig. 3:**
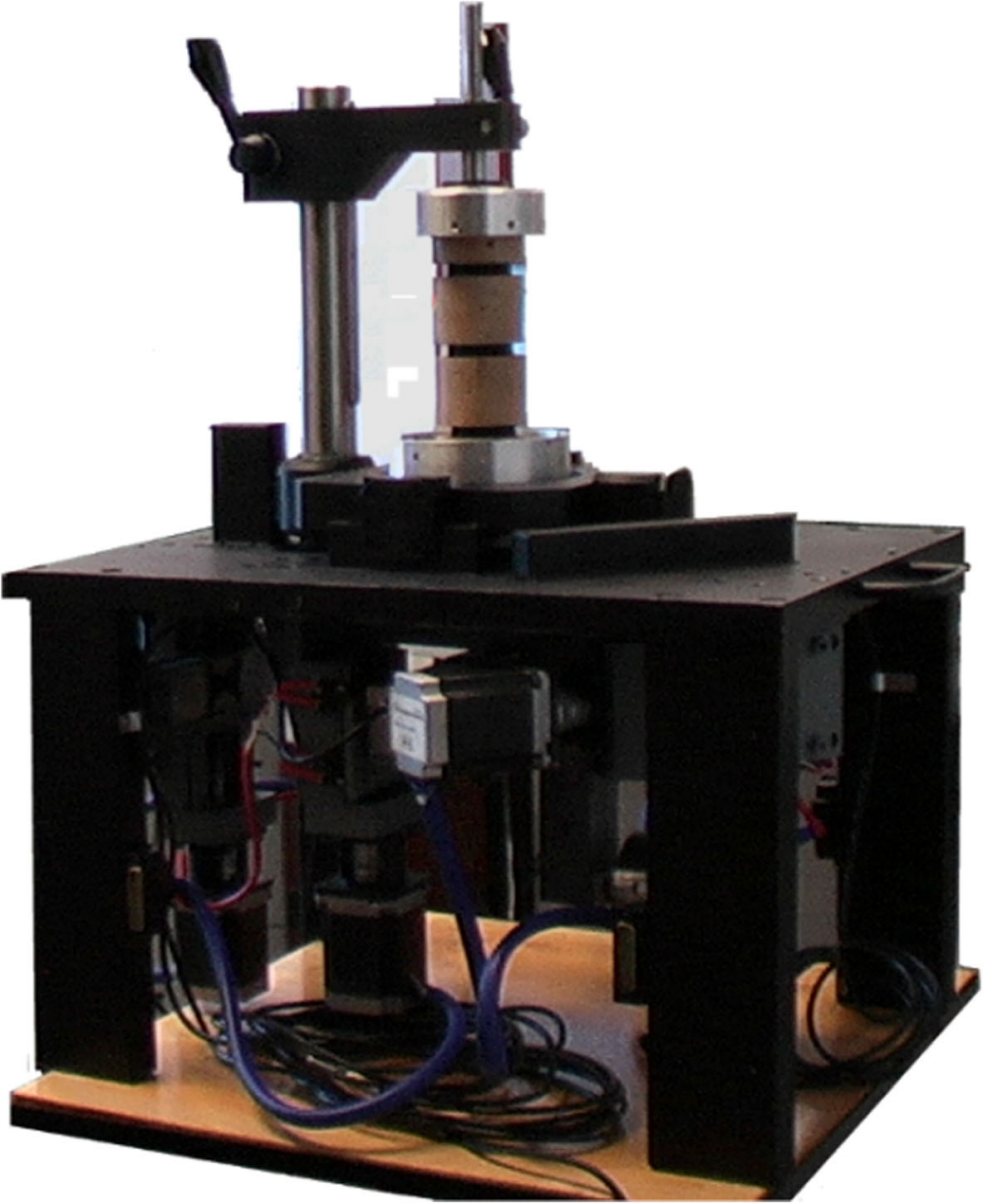
Second-generation BS-II—general view

**Fig. 4:**
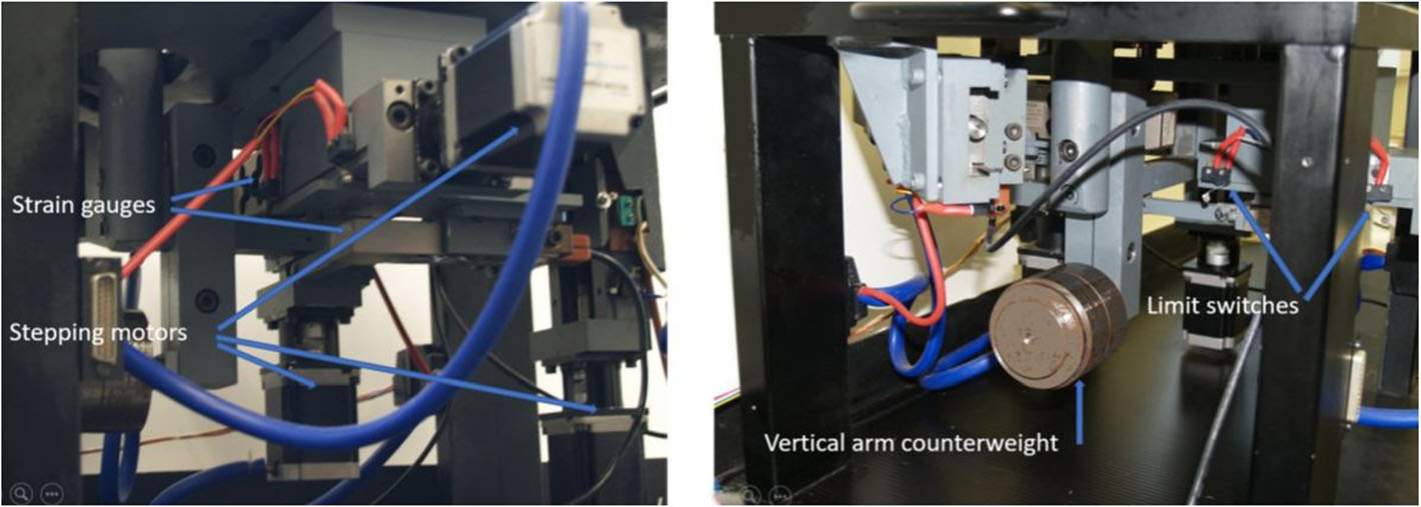
Second-generation BS-II—detail of the lower part of the machine

To ensure repeatability and statistical processing, a second-generation system BS-II was designed. The basis of the machine chassis is a massive metal plate, standing on four metal legs (Fig. 3), including mounting holes for individual components. The center of the construction passes through a system of levers and drawers, which together with the clamping system and four step-motors ensure movement of the sample during the measurement (Fig. 4).

**Fig. 5:**
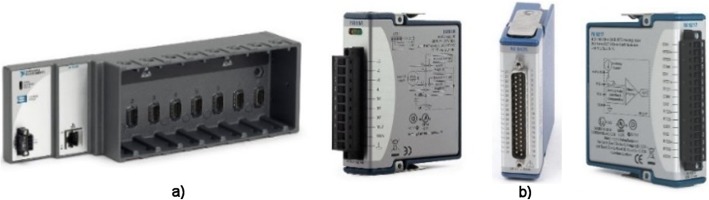
NI components. **a** Bus NI 9148. **b** NI CompactRIO modules

Measurement of flexural rigidity (front bend) and extension (rear bend), and right and left lateral bend (sample bend) is ensured by swinging the clamping system at the top and bottom of the machine. The left axial torsion (rotation) and axial compression (pressure) of the specimen ensure the lower part of the machine. The force required to handle the specimen is secured by four stepping motors and continuously captured by four strain gauges. To monitor the displacement of the vertebral vertebrae during the measurement, the sample is continuously captured by two perpendicularly fixed cameras. This improved construction of the measuring device makes it possible to perform the required biomechanical measurements of the sample in all the monitored axes without drawing it out. The entire device is controlled by the modern graphical programming environment LabVIEW, from National Instruments (NI) designed to the creation of so-called virtual measuring devices (Fig. 5a, b). Custom component control is done with the CompactRIO modular system. The NI 9148 chassis features 100MBit Ethernet interface and 8 slots for C Series modules. It includes the RISC processor and the NI VXWorks operating in real-time system. Measuring and collecting data are controlled via a personal computer (PC) via Ethernet. The NI 9148 chassis is powered by a 24-V DC NI PS-15.

**Fig. 6:**
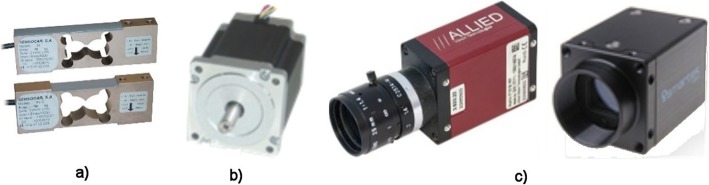
**a** Sensocar type BL-C strain gauges. **b** Stepping motors SX23-1020. **c** Vision MarlinF131B and Smartekvision GC1621MP cameras

The CompactRIO Module NI 9501 was used to control the two-phase stepping motors. It is capable of controlling motors in both unipolar and bipolar circuits. In our device, NI 9425 is a CompactRIO module with 32 digital inputs and is used to process signals from the limit switches of individual axes. The CompactRIO NI 9217 CompactRIO module can be used to process signals from thermistors and strain gauges operating in bridging. It includes four independent differential inputs and a 24-bit A/D converter with special electronics to reduce interference from the 50/60-Hz power grid. For noise suppression, strain gauges are powered from an external power supply with an integrated stabilizer 7815. For measuring force during flexion, extension and lateral bending are used four beams with Sensocar strain gauge type BL-C with a maximum load of 200 N and a sensitivity of 2 mV/V (see Fig. 6a). A similar type of strain gauge is used to measure pressure for loads up to 1000 N. The outputs of all strain gauges are outputs to the differential inputs of the 24-bit A/D converter of the NI 9217 measuring card.

**Fig. 7:**
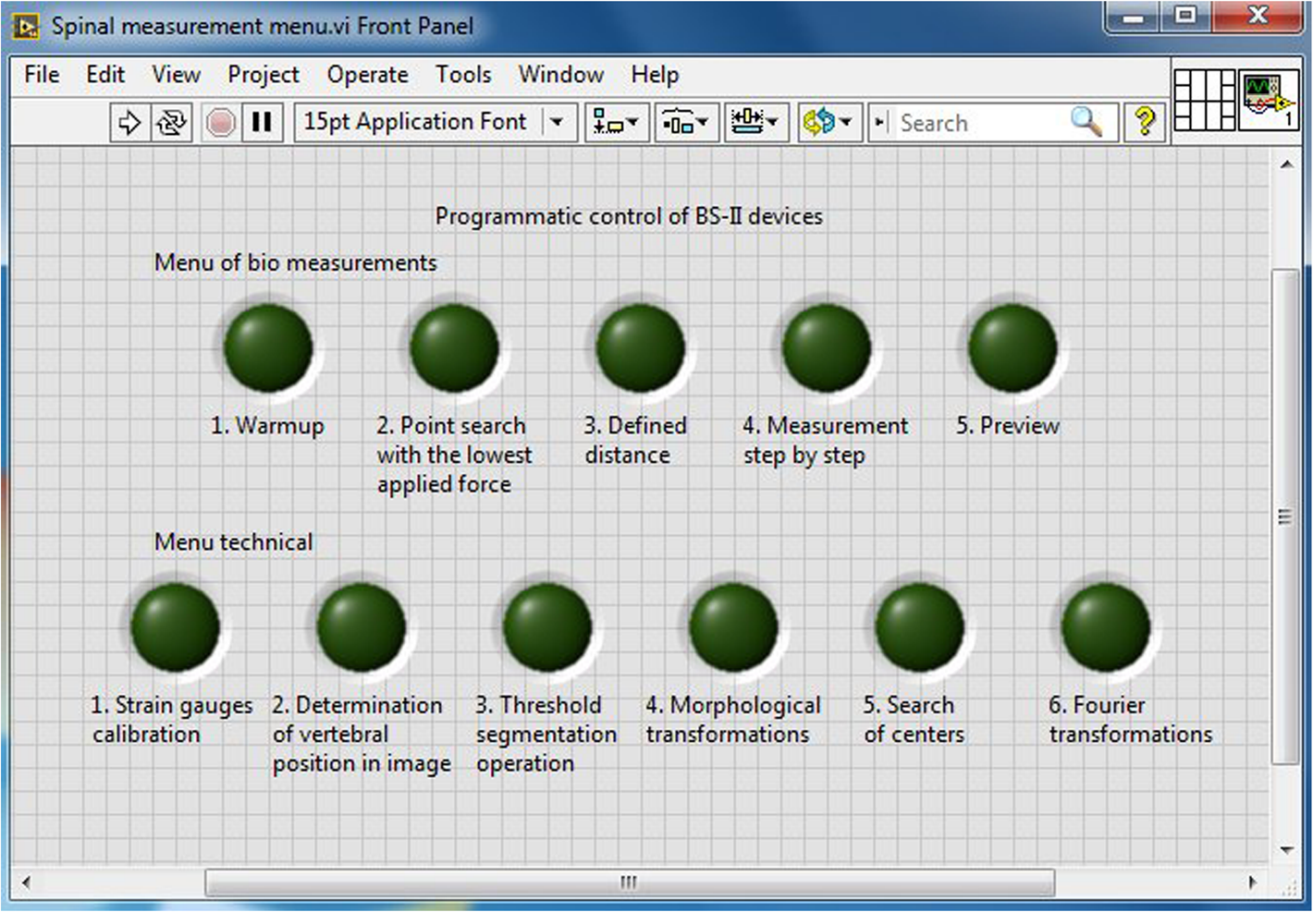
Program menu

The SX23-1020 two-step unipolar stepper motors from Microcon are used to move the sample (Fig. 6b). The motors are powered by NI 9501 control boards. Two cameras—Vision Marlin F131B from Allied Technology and Smartekvision GC1621MP—are used to capture images (Fig. 6c). All machine control is created in the LabVIEW programming environment and their libraries including system drivers for control hardware. Part of the created programs runs directly on the CompactRIO industrial computer (especially programs that access directly to the modules and their metering inputs/outputs), but their front panel is controlled by the PC. The second part of the programs containing more demanding software operations runs directly on the control PC. These two parts communicate with shared variables together. The program offers the following program modules to the user (see Fig. 7):

*Warmup (B1).* See button Menu Bio1. Prior to measuring, it is needed that the spine is being warmed up for achieving the defined physical parameters and to remove rigidity. This warming is done by circular movements in the *x* and *y* axes. The program code for the warm up before the measurement runs directly on the CompactRIO computer; the front panel is controlled from the PC.

*Point search with the lowest applied force (B2)* is running on the CompactRIO computer, the front panel is controlled by the PC. The program repeatedly measures the voltage at the strain gauge output and, accordingly, triggers the motors and moves the measured sample to the point where the lowest force is applied.

*Defined distance (B3)*. The program code for the defined distance travel runs on the CompactRIO computer; the front panel is controlled by the PC. The program is used to enter the exact number of steps in the specified direction. The number of steps is entered numerically, using the softkeys to select the axis and the direction of movement. The program then executes the specified move on a defined number of steps using the for cycle.

*Measurement step by step (B4).* The program is used for automatic measurement in steps. The number and length of the steps can be entered on the front panel. At each step, several strain gauge values are subtracted and statistically averaged to eliminate noise. The number of averages is adjustable. Next, it is necessary to setup the axis in which we want to measure (*x*, *y*, pressure, torsion) and set whether we want to measure from the starting point on both sides or just on one side from the starting point. In the program, you can choose whether to save photos from the measurements and the directory where the photos are saving. Measured data are displayed in arrays, which can easily be exported to the system clipboard or Excel.

*Preview (B5)*—camera adjustment program. The code for this program runs on PC only. The program is used to preview the cameras before starting the measurement itself. The preview of the cameras can be viewed either in one-shot way or it can be repeatedly updated in a loop. With this program, we can verify that the cameras are working properly, that their aperture is correctly set, and that their position is adjusted for accurate capture of the measured samples.

*Strain gauges calibration (T1).* See button Menu technical T1*.* Equations (4–7) show calibration of the strain gauges used for measuring force. This is necessary for voltage translate from strain gauges to units of force (Newton). The functional dependence of variables is solved by regression analysis expressed by the equation:

4 $$ y=a+ bx. $$

For linear regression, we used the “least squares method,” when coefficients *a* and *b* are calculated according to Eqs. (5) and (7), where all sums are taken from 1 to *n*:

5 $$ a=\frac{\sum {x}_i^2\sum {y}_i-\sum {x}_i\sum {x}_i{y}_i}{n\sum {x}_i^2-{\left(\sum {x}_i\right)}^2}=\frac{1}{n}\left(\sum {y}_i-b\sum {x}_i\right) $$

6 $$ a=\frac{\sum {x}_i^2\sum {y}_i-\sum {x}_i\sum {x}_i{y}_i}{n\sum {x}_i^2-{\left(\sum {x}_i\right)}^2}=\frac{1}{n}\left(\sum {y}_i-b\sum {x}_i\right) $$

7 $$ b=\frac{n\sum {x}_i{y}_i-\sum {x}_i\sum {y}_i}{n\sum {x}_i^2-{\left(\sum {x}_i\right)}^2}. $$

The calibration helps to decrease the measurement error below 5%. The shift of vertebrae has been tested employing the spine model using two cameras, see [12, 13]. The proposed arrangement confirms its functionality and is used to measure the biomechanical characteristics of real samples.

*Determination of vertebral position in digital image (T2).* Image processing requires a suitable mathematical tool that makes it easy to perform image operations. Basic methods of working with the image function operate with the pixel analysis and its particularly selected environment. The value of this pixel (in our case, the brightness) is then replaced by a linear combination of values in the area under investigation. The convolution is an important tool, which is defined in Eq. (8) for the continuous functions *f* (*t*) and *h* (*t*):

8 $$ g(t)=\underset{\tau =0}{\overset{+\propto }{\int }}f\left(\tau \right)\ h\left(t-\tau \right) d\tau =f(t)\times h(t). $$

The operator “×” indicates the symbolic relationship between the two functions *f* and *h* (the actual multiplication applies to Laplace images of these functions). The function *h* is also called a convolutional nucleus. For a discrete 2D function, the given relationship can be rewritten in in the shape (9):

9 $$ g\left(x,y\right)=\sum \limits_{i=0}^{M-1}\sum \limits_{j=0}^{N=1}f\left(i,j\right)\ h\left(x-i,y-j\right)=f\left(x,y\right)\times h\left(x,y\right), $$

where *M*, respectively *N*, is the number of columns, respectively (*i*, *j*) = 0. The convolution core *h* is discrete in the shape of a two-dimensional matrix and is called the filter. In our case, the Sobel matrix was used (10). It can reveal fine structures in galaxy arms. It calculates two convolution matrices along the *X* and *Y* axes. It can detect fine structures in the galaxy arms and calculate two convolution matrices along the *X* and *Y* axes.

10 $$ {\displaystyle \begin{array}{cccccc}1& 1& 1& 2& 2& 2\\ {}2& 2& 2& 2& 2& 2\\ {}3& 3& 3& 1& 1& 3\end{array}} $$

**Fig. 8:**
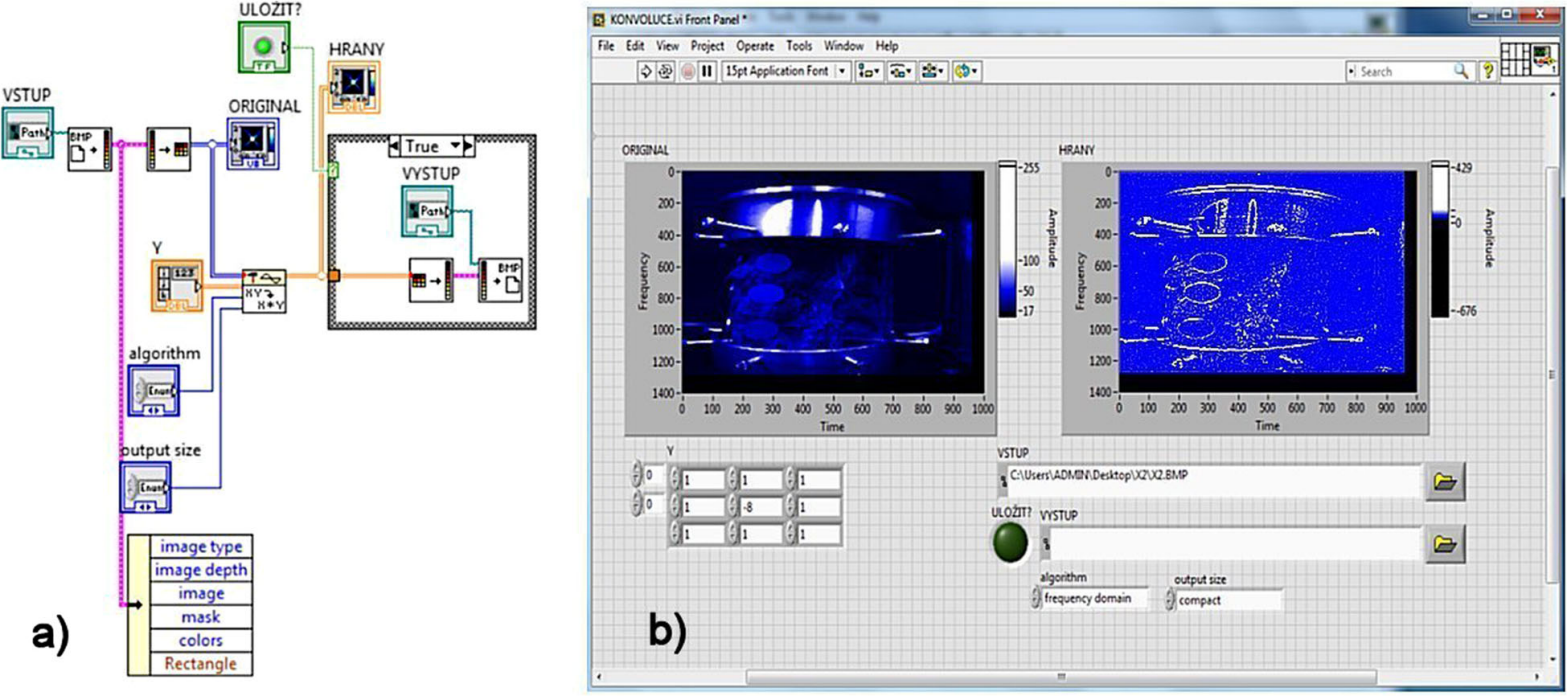
**a** Program convolution in LabVIEW. **b** Front panel of LabVIEW

This double convolution was used to process the image. Program implementation of convolution in LabVIEW is shown in Fig. 8a. LabVIEW offers a variety of features including basic imaging with image function. The Convolution.vi function is located on the subfolders Functions\Signal Processing and Signal Operations, and has two *x* and *y* signal inputs which we associate the imaging function of the convolutional core (filter) *h*. The *f* (*x*, *y*) image function is in the form of two dimensional matrices with the brightness values of individual pixels, for example, from a camera such as BMP.

Figure 8b shows a front panel of program LV, where the initial image (spine) is shown on the left, which is inserted in the form of a picture matrix. On the right, there is the result of the convolutional operation image with emphasized edges, which is the place of the maximum brightness change in the image corresponding to the second derivative. The following is a *Threshold segmentation operation (T3).* In the complete segmentation of the image R, we call the final set of areas {*R*_1_, *R*_2_, …, *R*_*s*_} for which it applies:

11 $$ R=\underset{i=1}{\overset{S}{\cup }}{R}_i,{R}_i\cap {R}_j=\varnothing \kern0.5em \mathrm{where}\ i\ne j. $$

Threshold is the transformation of the input image *f* to the output (segmented) binary image *g* according to the relationship:

12 $$ g\left(i,j\right)={\displaystyle \begin{array}{c}1\ \mathrm{where}\ f\left(i,j\right)\ge T,\\ {}0\ \mathrm{where}\ f\left(i,j\right)<T,\end{array}} $$

where *T* is a predetermined constant called a threshold and for image elements that belong to the segmentation of objects and *g*(*i*, *j*) for the background element which applies a threshold to the image based on the min and max threshold values that you enter. All pixels, not set between the minimum and maximum values, are set to 0. All pixels that fall in range are replaced by 1.

**Fig. 9:**
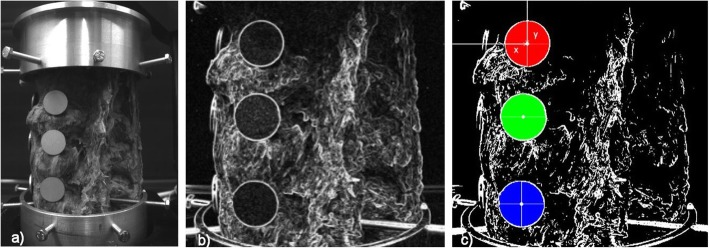
**a** Specimen photo of the spine. **b** Image transformation after convolution, segmentation, and morphology. **c** Determination of the centers of the circular discs

*Morphological transformations (T4)* are realized as an image relation (point set *X*) with another smaller set *B*, which is called the structural element. Operation used opening is an erosion operation followed by a dilation operation and is used to remove detail in the image that is smaller than a structural element, and the overall shape of the object is not broken (Fig. 9b). The opening of the set *X* by the structural element *B* is denoted*X* ∘ *B* and defined by the relation:

13 $$ X\circ B=\left( X\varTheta B\right)\oplus B, $$

where the symbol *Θ* indicates an erosion operation that can be entered in the form:

14 $$ X\varTheta B=\left\{d\in {E}^2:d+b\in X\kern0.5em \mathrm{where}\forall b\in B\right\} $$

and the symbol ⊕ indicates a dilatation operation with an expression:

15 $$ X\oplus B=\left\{d\in {E}^2:d=x+b,x\in X,b\in B\right\}. $$

*Search of centers (T5)*. The program goes through the image at the *x* and *y* coordinates and, in the case of the specified color, calculates the average of the individual rows and columns, which average a value of the *x* coordinate and the value *y*. This value determines the center of the corresponding circular discs in the monitored image. The values *x* and *y* are stored in the memory. See Fig. 9c.

*Fourier Transform (T6)*. To detect small movement changes, a modified speckle technique is proposed (Fig. 13a–c) where the pseudo-speckled specimens, generated in a standard deviation computer*σ* = 50, are scanned by CCD camera before and after shifting and evaluated using a 2D FFT according to the Eq. (16). See [12]:

16 $$ F\left(x,y\right)={F}^{-1}\left\{f(t)\right\}=\frac{1}{2 pi}\underset{-\propto }{\overset{\propto }{\int }}\underset{-\propto }{\overset{\propto }{\int }}F\left(\varsigma, \xi \right){e}^{-i\left( x\varsigma + y\xi \right)}\mathrm{d}\varsigma \mathrm{d}\xi . $$

**Fig. 10:**
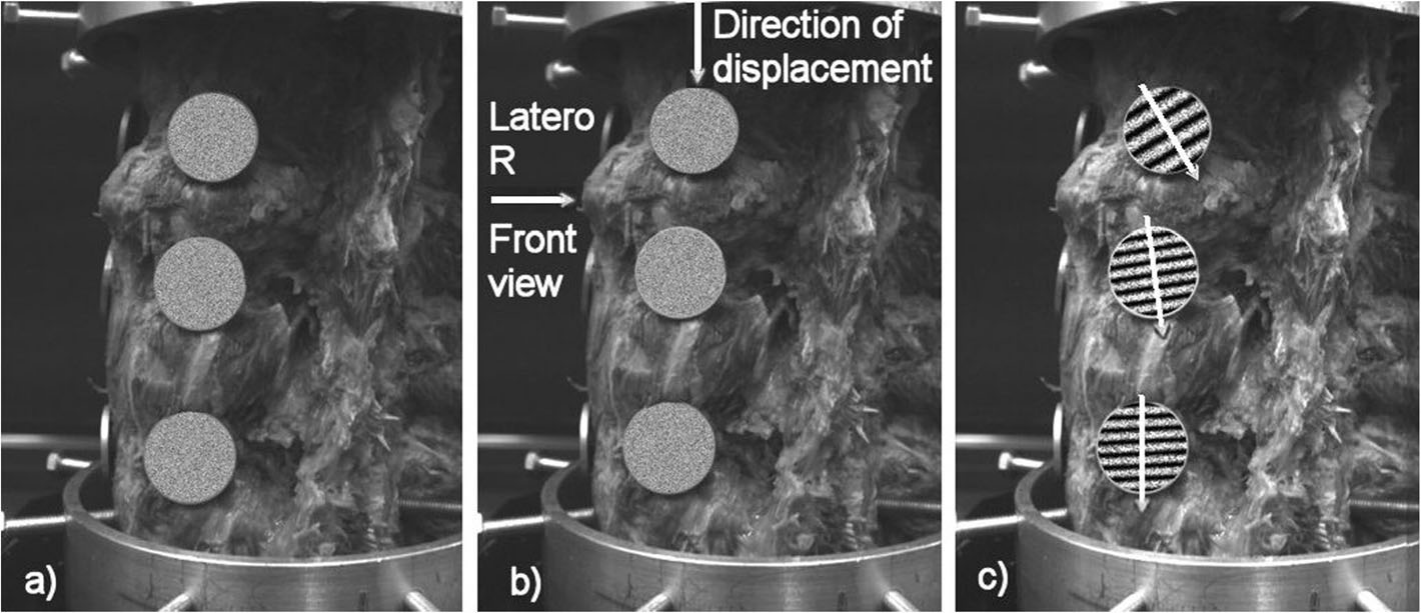
**a** Targets covered pseudo-speckled specimens (*σ* = 50) and connected to the specimen of the spine. **b** Circular discs displacements. **c** Fourier spectrum of the sum of two consecutive image recordings in the measurement of flexion and the directions of movement of the circular discs

The obtained Fourier picture (Fig. 10c) of the spectrum of the picture (Fig. 10b) is formed by interference strips, where the respective spatial frequency (strip density), resp. the period determines the target displacement size, and the pitch of the interference strips determines the direction of the target shift (Fig. 10c). Images are captured by two cameras due to their spatial display. See the block diagram in Fig. 1.

### Measurement of specimen

**Fig. 11:**
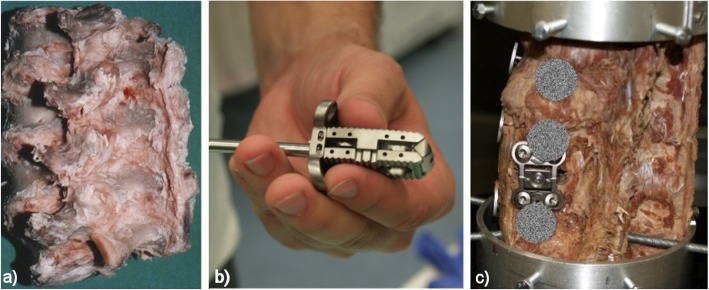
**a** Human cadaverous spine sample. **b** Implant LUMIR XLIF CAGE. **c** Application of implant on cadaveric specimen

A unique intervertebral implant (Fig. 11b) has been developed at the neurosurgical clinic of the Faculty Hospital Olomouc and the Faculty of Medicine, Palacký University. It is the first type of substitution able to adapt to the specific size of the intervertebral space. The advantage of a new implant is its material (titanium alumina, alumina, and vanadium), which exhibits primary stability from the moment of introduction [28–31].

For the realization of the experiment, a cadaverous sample of the human lumbar spine was obtained from Faculty Hospital Olomouc (Fig. 11a). The sample was completed with two aluminum jigs which, by means of self-tapping hexagon, bolt it fixed firmly in both axial and radial directions with L1 and L5. Mechanically fixed sample is inserted into apparatus type BS-II and, by means of additional mechanical elements, is firmly connected to parts of the apparatus which are adapted to the individual types of a sample loading. Measuring circular discs were attached to the monitored parts of the sample. The unadjusted (intact) spine sample was warmed up at frequency of 0.1 Hz [1]. Subsequently, the values were stored of the instant rigidity of the flex (front bend), extension (back bend), right and left lateral flexion (lateral bend), left and right axial torsion (rotation) and axial compression (pressure), and pictures from both cameras in individual positions. This measurement sequence was repeated 10 times, and the data obtained were stored on a suitable data medium. Subsequently, the participants of the medical expertise team implemented a unique LUMIR XLIF CAGE implant (Fig. 11b, c). The stabilized sample was measured in the same manner and under the same conditions (heat, laboratory humidity). Instant rigidity and camera images have been saved. Comparison of the rigidity of the intact and stabilized sample is shown in the following graphs. The cameras record the movement in two orthogonal directions and the movements of the parts of the sample, respectively their circular discs, i.e. vertebrae L2, L3, and L4.

## Results

**Fig.12:**
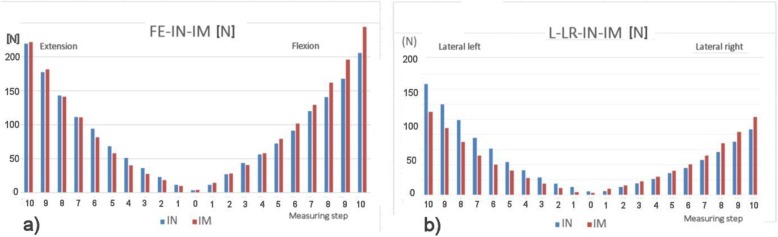
**a** Flexion-extension instant rigidity graph int. and impl. sample. **b** Lateroflexion graph of instant stiffness at int. and impl. sample

**Fig. 13:**
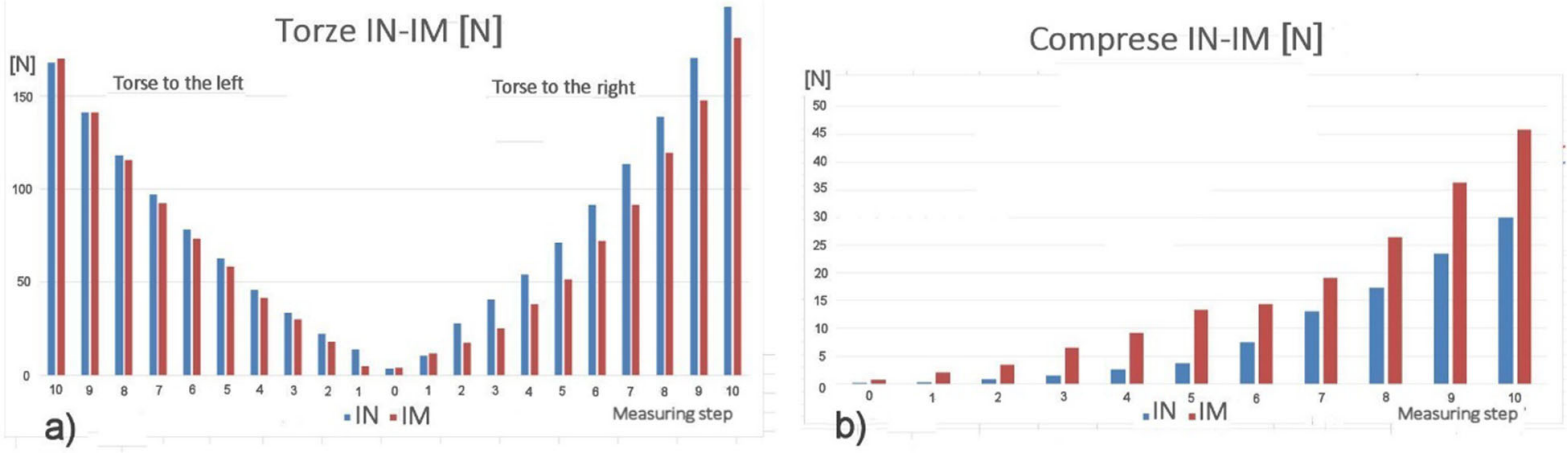
**a** Graph of instant rigidity in torsion (L, R) int. and impl. sample. **b** Graph of pressure instant rigidity of int. and impl. sample

The results of the measurement of the instant rigidity of the intact sample and stabilized sample for the individual measurements are clearly recorded in Figs. 12 and 13.

**Fig. 14:**
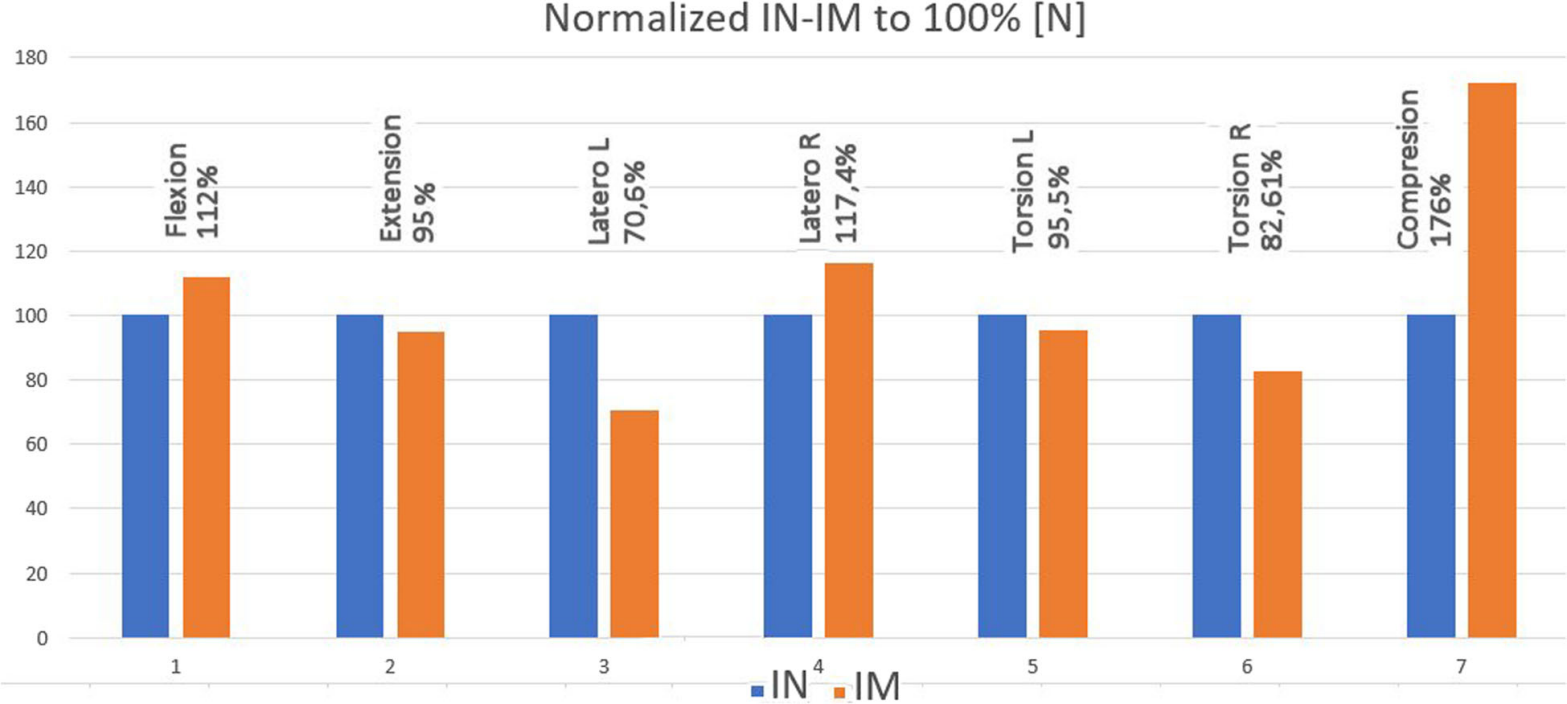
Normalized graph IN-IM 100% [N]. The orientation as shown in Fig. 1

For the immediate overview, the graph in Fig. 14, containing the results related to the intact spine (1.00 to 100%), is attached and is therefore normative.

**Fig. 15:**
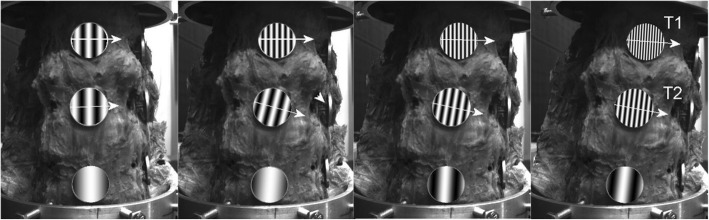
Direction of movements of individual vertebrates at intact sample, right side bend, direct view

**Fig. 16:**
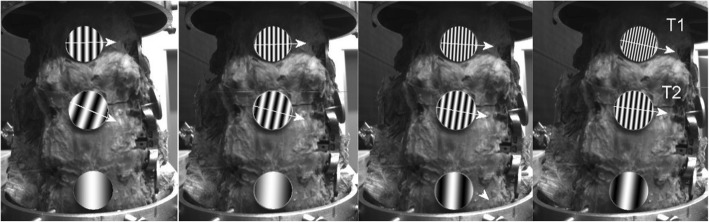
Direction of movements of individual vertebrates at sample with implant, right bend, direct view

The device also allows the analysis of the movement of the individual components of the spinal sample (see Figs. 15 and 16). As an example, the movements of the individual components are documented in the front view for the left bend for the intact specimen and the specimen with the LUMIR XLIF CAGE implant. The displayed changes in the movement of each component are consistent with the expected changes due to implant insertion.

## Discussion

The new second-generation computer system BS-II based on the National Instruments’ development environment was used to measure the biomechanical properties of the implant, instead of its manually operated ancestor [13]. The system allows circular movement (warm up of the specimen) and programmatically controlled extension (back bend), right and left lateral flexion (lateral bend), left and right axial torsion (rotation), and axial compression (pressure). The system further measures the stiffness of the sample and the movement of individual components (vertebrae). When testing the implant implantation in a human dead sample, we have discovered that the sample of cadaverous spine stabilized by LUMIR XLIF CAGE implant reveals an extension resistance of less than 95% and greater resistance to flexion of about 112% than for extension, which is logical, just as is logical of considerably greater resistance to compression. It also showed an interesting lateral flexion with implant in T1 and the smaller resistance in T2, which is logically depending on the location of the implant. The change of the right-hand side is described as shown from the point of view of Fig. 1, because the implant is inserted from the left. The functionality and accuracy of the BS-II system was verified on mechanical models of the spine and plastic models created by 3D printing [14]. Due to strict legislation, only one cadaveric sample of the human lumbar spine was used in the BS-II series of functional testing.

## Conclusion

The designed computer BS-II system is designed for the verification of surgical fixation methods of the spine. The system is based on the knowledge and experience with a manually operated measuring device designed by Palacky University Olomouc [12, 13]. If appropriate models are used (3D print), the BS-II system can be used to verify surgical procedures of stabilizing the spine while teaching future doctors. The usability of the BS-II system is demonstrated on the case of the implantation of LUMIR XLIF CAGE implant to a human cadaver specimen of the spine.

### Postscript

From December 2017 to June 2018, 24 implants have been implanted into 21 patients according to the number of the pathological discs (in 2 patients, 2 implants in 2 segments were implanted, and for the remaining patients—just one implant).

## Data Availability

All measured data are freely available in this article, and there are no additional datasets outside.

## Abbreviations

ALIF: Anterior lumbar interbody fusion
BS-II: Bio-spine second-generation measurement device
CCD: Charge couple devices
DIP: Digital image processing
FFT: Fast Fourier transformation
NI: National Instruments
XLIF: Extreme lateral interbody fusion
